# The small molecule Mek1/2 inhibitor U0126 disrupts the chordamesoderm to notochord transition in zebrafish

**DOI:** 10.1186/1471-213X-8-42

**Published:** 2008-04-17

**Authors:** Thomas A Hawkins, Florencia Cavodeassi, Ferenc Erdélyi, Gábor Szabó, Zsolt Lele

**Affiliations:** 1 Department of Anatomy and Developmental Biology, University College London, Gower Street, London, WC1E 6BT, UK; 2 Department of Gene Technology and Developmental Neurobiology, Institute of Experimental Medicine, Hungarian Academy of Sciences, Szigony u. 43. Budapest, H-1083, Hungary

## Abstract

**Background:**

Key molecules involved in notochord differentiation and function have been identified through genetic analysis in zebrafish and mice, but MEK1 and 2 have so far not been implicated in this process due to early lethality (*Mek1-/-*) and functional redundancy (*Mek2-/-*) in the knockout animals.

**Results:**

Here, we reveal a potential role for Mek1/2 during notochord development by using the small molecule Mek1/2 inhibitor U0126 which blocks phosphorylation of the Mek1/2 target gene Erk1/2 *in vivo*. Applying the inhibitor from early gastrulation until the 18-somite stage produces a specific and consistent phenotype with lack of dark pigmentation, shorter tail and an abnormal, undulated notochord. Using morphological analysis, in situ hybridization, immunhistochemistry, TUNEL staining and electron microscopy, we demonstrate that in treated embryos the chordamesoderm to notochord transition is disrupted and identify disorganization in the medial layer of the perinotochordal basement mebrane as the probable cause of the undulations and bulges in the notochord. We also examined and excluded FGF as the upstream signal during this process.

**Conclusion:**

Using the small chemical U0126, we have established a novel link between MAPK-signaling and notochord differentiation. Our phenotypic analysis suggests a potential connection between the MAPK-pathway, the COPI-mediated intracellular transport and/or the copper-dependent posttranslational regulatory processes during notochord differentiation.

## Background

One of the greatest challenges in developmental biology is to bridge the gap between cell biology and experimental developmental genetics (ie. to link the function of a protein at the level of cell and organism). In order to achieve this, one has to use the methods, tools and results offered by other research fields. For developmental biologists, one possibility is to start *in vivo *testing of small molecules identified in chemical array experiments once their specificity is satisfactorily established in biochemical and cell culture assays. The use of such specific chemicals could identify functions of a protein obscured by early lethality in knockout or transgenic animals or by functional redundancy due to the activity of paralogous genes. This approach is also attractive as small molecules/drugs can be applied and withdrawn at will, providing an alternative for expensive and time-consuming transgenic experiments. The use of signaling pathway modifying chemicals is particularly feasible in classic genetic model organisms such as Drosophila and zebrafish, due to their relative cheapness and the availability of large numbers of externally and quickly developing embryos which allows rapid and parallel testing of various concentrations and application time points [1]. Recently several chemicals have been tested which are now widely used as inhibitors of certain pathways in developmental studies (eg. SU5402-fibroblast growth factor (FGF) signaling pathway, cyclopamine-hedgehog (Hh) signaling pathway, SB-431542-TGFβ signaling pathway [2-6]). Moreover, large-scale small molecule screens have been carried out to identify potential drugs for various diseases [7,8].

The compound U0126 (1,4-diamino-2,3-dicyano-1,4-bis [2-aminophenylthio]butadiene) was originally identified as an inhibitor of AP-1 transactivation in a cell-based reporter assay [9]. This inhibition turned out to be due to direct and specific inhibition of the mitogen-activated protein kinase kinase (MAPKK) family members, MEK1 and MEK2. The MAPK pathway is one of the most thoroughly characterized intracellular signaling pathways transmitting extracellular signals (eg. growth, stress or differentiation factors) [10-12]. It has been implicated in various processes including cell proliferation, survival and differentiation [13] as well as in development [14]. Currently there are 6 known MAPK signaling pathways: (ERK1/2, ERK3/4, ERK5, ERK7/8, JNK1/2/3 and p38/ERK6) and although *in vitro *studies have described biochemical characteristics of these cascades in detail, their diverse (or redundant) roles during vertebrate development have only recently come under scrutiny [15-17]. Inhibitory activity of U0126 is selective for MEK1 and MEK2, and shows very little, if any, effect on the kinase activities of other protein kinases like c-Abl, Raf, MEKK, ERK, JNK, MKK-3, MKK-4/SEK, MKK-6, Cdk2, or Cdk4 [18]. Since its description, more than 1500 papers have used this inhibitor, confirming its specificity *in vitro*. Results of *ex vivo *tissue explant experiments have implicated the involvement of MEKs in a wide range of developmental processes including angiogenesis [19,20], renal tubulogenesis [21,22], somitic segmentation [23], lens differentiation [24] as well as guidance and segregation of retinal afferents during mammalian visual system development [25,26]. *In vivo *testing of U0126 has been carried out in ascidian species (Halocynthia roretzi and Ciona intestinalis), where U0126 treatment blocked differentiation of mesenchyme, secondary muscle and neural tissues and formation of the notochord (NC) [27-29].

The NC serves as the most important skeletal structure in lower chordates and plays an essential role in vertebral column development in vertebrates. Its equally important function is to provide critical signaling molecules to neighbouring tissues (eg. neurectoderm, paraxial mesoderm), directing their differentiation [30]. The mature NC develops from the chordamesoderm, a derivative of dorsal mesoderm, and is ultimately incorporated into the forming vertebrae as the nucleus pulposus.

Here we report the analysis of zebrafish embryos treated with the MEK1/2 inhibitor U0126 which causes an almost 100% penetrant, dose-dependent and reproducible phenotype consisting of short trunk and tail, lack of dark pigmentation, and abnormal NC development. Time-course and washout experiments revealed that the treatment has to be applied within a strict time window from the beginning of gastrulation until 16–18-somite stage (around 18 hours post-fertilization, hpf) and the strength of the phenotype correlates with the level of Erk phosphorylation observed in the embryos. The NCs of U0126 treated embryos develop undulations and form multi cell-layer lumps instead of the single „stack-of-coins" structure observed in wild type siblings. Electron microscopy revealed defects in the multilayered structure of the perinotochordal basement membrane (PNBM) which supports NC cells against their own high internal hydrostatic pressure. We show that the shorter tail of the treated embryos is due to an increase in apoptosis and not to a decrease in number of mitotic cells. Gene expression analysis showed that U0126 affects the chordamesoderm to mature NC transition step. Surprisingly, ventral fates in the neural tube are not affected while development of the dorsal aorta and intersomitic vessels is severely disturbed. U0126-treated embryos do not show any significant similarity to those treated with the FGF-signaling inhibitor SU5402 which suggests that an alternative pathway is required to activate MEK during NC development. We also examined possible links to pathways whose inhibition in different mutants produce a very similar phenotype including COPI-mediated intracellular transport and the copper-transporter Atp7a. We conclude that MEK1/2 inhibition did not decrease the expression of any of the potential genes examined, nevertheless, interactions between these processes and MAPK1/2 signaling may exist at the protein level.

## Results

### U0126 treatment during gastrulation causes notochord and pigmentation defects in zebrafish

**Figure 1:**
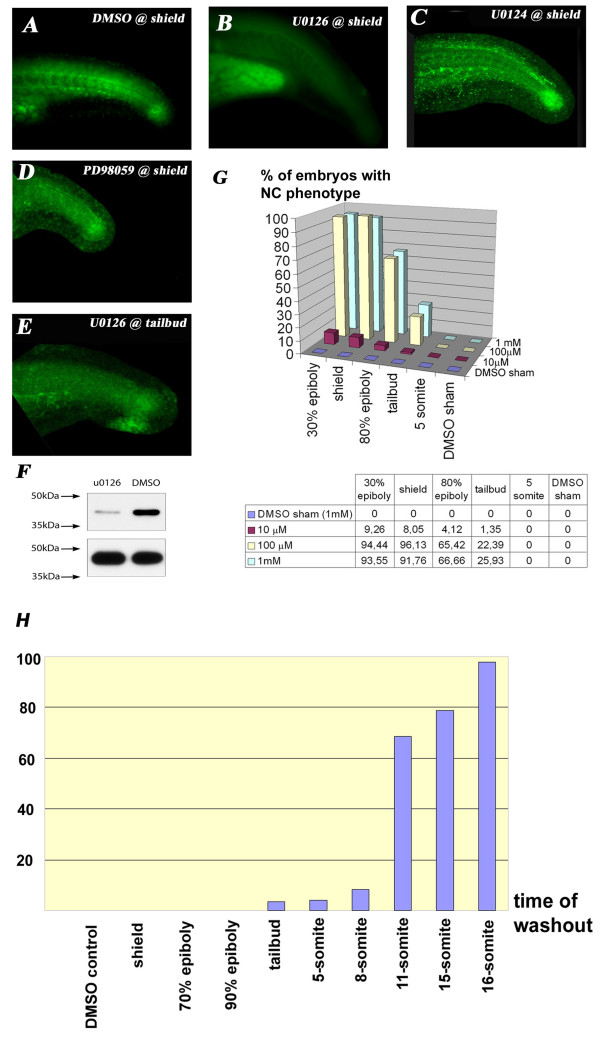
**U0126 application during a specific time-window decreases ERK phosphorylation specifically**. **A**: Diphospho-Erk staining in the trunk of wt 24 hpf embryos.**B**: U0126 eliminates ERK1/2 phosphorylation (green) in the trunk and tail of 24 hpf zebrafish embryos. Notice that U0126-treated embryos have been overexposed (can be judged by the autofluorescence of the yolk sac extension) and still fail to show specific staining. **C**: U0124 does not eliminate the p-Erk staining. **D**: PD98059 also does not eliminate p-Erk staining when given just below the level of lethal toxicity (25 μM). **E**: Somewhat weaker but still significant p-Erk staining in embryos treated with U0126 starting at 10 hpf. **F**: Western-blot analysis demonstrated a slight but incomplete reduction in pErk1/2 levels in 10-somite embryos.**G**: Stage and dose-dependency of U0126 application. There is a dramatic drop in the percentage of affected embryos when treatment is applied after epiboly is completed. **H**: Washout experiments reveal the necessity of U0126 to be present until at least the 18 somite stage to produce the full phenotype.

The specificity of U0126 as a MEK1/2 inhibitor has already been established by many studies both *in vitro *[31] and *in vivo *[32] including one analysis in zebrafish [33]. Consistent with those analyses, treatment of zebrafish embryos with U0126 from early gastrulation led to complete abrogation of the phosphorylation of Erk1/2, the downstream target of Mek1/2 (Fig. 1/A,B). This was not the case when the embryos were treated under the same the same conditions with U0124, a small molecule with a slightly changed composition which results in loss of MEK1/2 inhibitory capacity, supporting the specificity of the U0126 effect (Fig. 1/C). PD98059, also used as a MEK1/2 inhibitor *in vitro *[34], caused no phenotype whatsoever at 20 μM and resulted in 100% lethality at 30 μM concentration. PD98059 has been shown to be 100-fold less potent than U0126 *in vitro *[9]. This suggests that its lethality at such a low concentration was due to non-specific causes, preventing us from analysing its effect as a MEK1/2 inhibitor. Indeed, embryos treated with sublethal 20 and 25 μM PD98059 (Fig. 1/D; panel is 25 μM) had normal phospho-ERK1/2 staining, suggesting normal MEK activity.

Next we tested the inhibitor at several concentrations and at several starting time-points (Fig. 1/G). The lowest effective concentration that still gave the strongest observed phenotype (detailed below) was 100 μM, so that was used in all subsequent experiments. Higher concentrations of the inhibitor (up to 1 mM; U0126 precipitates beyond 1 mM) did not produce a stronger or different effect, suggesting low non-specific toxicity of U0126. The induced phenotype was very distinct and reproducible, while DMSO sham controls never showed any defects. In order to achieve maximum effect, it was necessary to apply the inhibitor during early gastrulation (Fig. 1/G). Under these conditions (100 μM applied at early gastrulation), the efficacy rate of the experiments never fell below 90% in several thousand embryos. When the drug was added at later stages (starting 85–90% epiboly or afterwards) the effect on Erk1/2 phosphorylation was proportionally weaker (Fig. 1/E), appeared later and affected only the posterior part of the NC. Treatment with lower concentrations of U0126 from early gastrulation also produced a weaker phenotype and reduced the number of affected embryos (Fig. 1/G).

To examine whether slow penetration of U0126 into the embryo might explain why there is such a delay between treatment and derived effect we stained embryos at 10 somite stage with pERK1/2 (as above). As suspected, there was no decrease in pErk1/2 signal (data not shown) suggesting that the drug did not penetrate quickly. To examine this with greater sensitivity we carried out Western-blot analysis using the pERK1/2 antibody on lysates of the U0126 treated and DMSO control embryos at the same 10 somite stage. This revealed a decrease in pErk1/2 after U0126 treatment (Fig. 1/F). Nevertheless, there was still some pErk1/2 present indicating slow penetration and/or effect of the drug in zebrafish embryos.

To identify the time point when U0126 has to be present to induce the NC phenotype, we also carried out washout experiments at different stages of development. These demonstrated that U0126 has to be continously present from the onset of gastrulation until at least 17–18 somite stage to achieve full penetrance of the phenotype (Fig. 1/H). When washed out earlier than that, only the anterior part of the NC was affected. This result, combined with the late application data (Fig. 1/G) suggests the existence of a time-window during which the NC cells are sensitive to U0126. This sensitivity window moves in an A-P direction as the NC differentiates. For subsequent experiments we routinely started treating the embryos at the onset of gastrulation and kept them in the inhibitor until the required fixation time-point.

**Figure 2:**
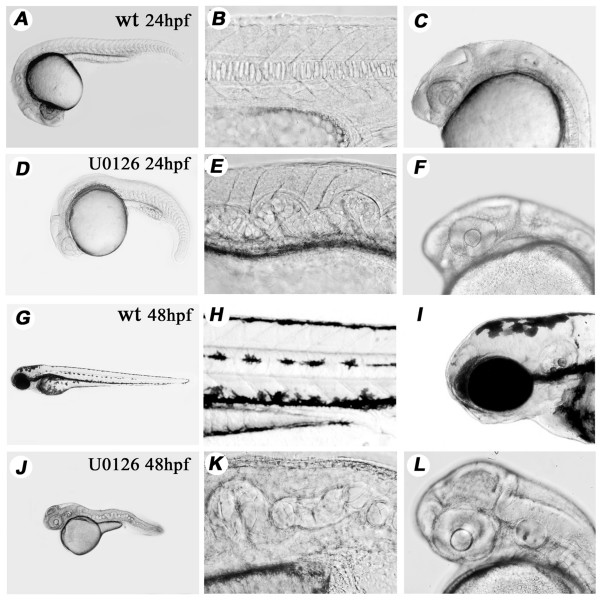
**U0126 produces a specific and consistent phenotype affecting pigmentation and notochord development**. Phenotype of wild type and U0126-treated embryos at 24 hpf. (**A-F**) and at 48 hpf (**G-L**). There is a total lack of pigmentation including in the RPE in the eye (compare panels **I **and **L**), twists and undulations in the NC are already evident at 24 hpf. (**D**, **E**) and the trunk and tail of treated embryos is shortened at 48 hpf (compare panels **G **and **J**). The large yolk sac in 48 hpf embryos indicates that circulation is not established properly to carry away its content (see also later in Fig. 5) Panels **A-C**, **G-I**: wild type embryos. Panels **D-F**, **J-L**: U0126-treated embryos.

U0126 incubation produced a phenotype with two major characteristics. First, the embryos lacked all accumulations of the dark pigment melanin and remained completely translucent provided that they were kept continuosly in the inhibitor (Compare Fig. 2/G–I with Fig. 2/J–L). If taken out from the U0126 solution after 24 hpf, dark pigmentation slowly recovered although it never became quite as strong as it was in control embryos. Following this recovery, the pattern of pigmentation was identical to the untreated controls', albeit not as strong. A likely explanation for the pigmentation effect of U0126 was provided recently by Gelfand and colleagues who showed that U0126 treatment inhibits melanosome transportation within fully differentiated melanophore cells *in vitro *[35].

**Figure 3:**
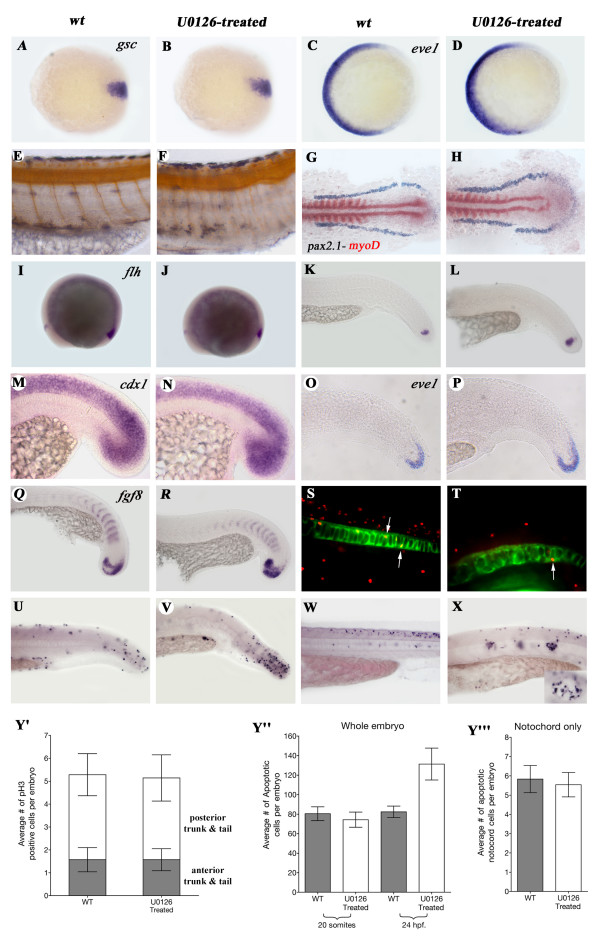
**Early processes are not affected by U0126 with the exception of apoptosis**. **A-D**: Expression the dorsal marker *gsc *(**A**, **B**,), and the ventral mesodermal marker gene *eve1 *(**D**, **E**) is not changed in 7 hpf. U0126-treated animals. As treated and wt animals could not be separated at this stage, a large number of embryos were treated and some of them were raised till 24 hpf. to make sure the drug was effective. **E**, **F**: Narrower, straighter somites in U0126-treated embryos as demonstrated by the reduced distance between the motoneurons (1 motoneuron/somite) stained with α-Acetylated-Tubulin antibody. **G**, **H**: Lateral mesoderm marker gene *pax 2.1 *(purple) and the somitic mesodermal marker gene *myoD *(red) do not show altered expression in dorsal view of flat-mounted 9-somite stage embryos. Expression of genes involved in tail development is not changed after U0126-treatment. **I-L:***flh*. M, N: *cdx1*. **O.P**: *eve1*. **Q**, **R**: *fgf8*. **S**, **T**: Examples of *shh-GFP *and α-phosphorylated Histone3 double antibody staining used to count mitotic cells in the NC. **U-X**: Examples of TUNEL stainings of 24 hpf (**U**, **V**) and 48 hpf (**W**, **X**) wt and U0126-treated embryos (inset in panel **X **shows larger magnification and see these cells also in **Fig. 6/F **marked with yellow arrows). **Y'**: The average numbers of phosphorylated Histone3 (pH3) positive cells in the anterior and posterior trunk and tail do not differ between wt and U0126-treated embryos. Average numbers of TUNEL positive cells in whole embryos (Y") and notochord-only (Y"') at 20 somites and 24 hpf demonstrate a significant increase in U0126-treated embryos at 24 hpf (p = 0.0087 unpaired t-test with welch's correction, 24 hpf values). Note that there is no change in apoptosis within the NC. Panels **A**, **C**, **E**, **G**, **I**, **K**, **M**, **O**, **Q**, **S**, **U**, **W**: wild type embryos. Panels **B**, **D**, **F**, **H**, **J**, **L**, **N**, **P**, **R**, **T**, **V**, **X**: U0126-treated embryos. Panels **A**, **D **animal pole view, dorsal to the right.

The second major defect consisted of severe morphological abnormalities in the trunk of U0126 treated embryos. The first sign of this was a ventral bending of the tail of treated embryos at around 20 hpf. By 24 hpf the the NC was severely affected (Fig. 2/A,B,D,E), while other parts of the treated embryo (brain, somites) appeared morphologically normal (Fig. 2/C,F). By 48 hpf the tail was significantly shorter (compare Fig. 2/G,J), the somites had a straight shape instead of the normal chevron (V) shape (see also Fig. 3./E,F), and the NC developed very severe kinks, thickenings and undulations, sometimes even knot-like features (Fig. 2/H,K). At this stage the structure of the brain still appeared normal (Fig. 2/I,L), but soon afterwards a necrotic process was initiated here which spread over the embryo resulting in death during the following couple of days.

### U0126 does not affect early dorso-ventral patterning and convergent-extension movements during gastrulation

The previous experiments revealed that in order to achieve the maximum NC phenotype, inhibitor treatment must commence at the start of gastrulation, so we examined whether patterning processes occuring at this time are affected by U0126. FGF signaling, which transduces its signal through the MAPK pathway, plays a significant role in zebrafish dorso-ventral (D-V) and anterior-posterior (A-P) patterning and tail formation [36-39]. Moreover, embryos of mildly dorsalized zebrafish mutants (eg. *swirl, somitabun, lost-a-fin, minifin, snailhouse*; [40,41]) have a shift in mesendodermal fate, producing more notochordal tissue and less, or no, ventral mesoderm which could be the reason for the undulating NC found in U0126-treated embryos. To examine this possibility, we checked distribution of *gsc *and *eve1*, a dorsal and ventral mesoderm marker, respectively. Neither of these (Fig. 3/A–D), nor similar markers examined (data not shown) had an altered expression pattern, indicating that D-V specification is not disturbed by U0126 treatment.

Another process that may be affected by U0126 treatment is convergent-extension (C-E). C-E defects produce a short trunk and compressed, straight somites similar to those observed in U0126-treated embryos (Fig. 3/E,F) [42,43]. Moreover, a C-E mutant *knypek *encodes the zebrafish homologue of Drosophila glypican: a heparan-sulfate proteoglycan mediator of FGF signaling [44]. However, 8-somite stage U0126-treated embryos showed no defects in the expression of somitic and lateral mesodermal marker genes (*myoD *and *pax2.1 *respectively), indicating no delays in C-E movements of mesodermal cells (Fig. 3/G,H).

Alternatively, the short tail phenotype could also be the result of a defect in tail elongation. However, no change was observed in the expression of a number of genes known to be involved in this process, such as *flh, fgf8, eve1 *or *cdx1 *(Fig. 3/I–R).

We next checked whether an increase in cell division in the NC could be responsible for the generation of undulations in this stucture. To follow changes in mitosis, we counted dividing cells in the NC of wild type and treated embryos stained for the mitotic marker phospho-histone3 (which labels cells that are in the M phase at the time of fixation). To ensure that we only counted notochordal cells, we used *shh-GFP *embryos [45] as a counterstain and only double stained cells were counted (arrows in Fig. 3/S,T,Y'). This revealed no differences in the number of M phase cells in the NC at 24 hpf in control *versus *U0126-treated embryos (Fig. 3/Y'; wt: 37 α-H3 positive NC cells/7 embryos; U0126-treated: 35 positive NC cells/7 embryos). To assess any changes in the A-P distribution of mitoses, cells between the posterior tip of the tail and the end of the yolk tube extension were also counted separately. The distribution of mitotic cells also did not change in treated embryos (11 cells/7 embryos in both cases). Thus, changes in cell division rates in the NC are unlikely to be responsible for its undulations. U0126 treatments did, however, increase apoptosis in the tail of treated embryos, as assessed by TUNEL staining. Since the number of TUNEL positive cells showed substantial variability (between 20 and 150 cells/embryo), we carried out counting on a large number of embryos (24 embryos/sample). At 20 hpf there was no difference in the number of apoptotic cells between wild type and treated embryos (Fig. 3/Y"), but by 24 hpf there was a specific and severe increase of apoptosis in the tail mesenchyme (Fig. 3/U–V,Y") of about one-third of the treated embryos. The increased apoptosis in the tail mesenchyme could explain the shortness of 48 hpf embryos, and may account, at least partially, for the undulations of the NC. On the other hand, we could not detect any increase in apoptosis within the NC (Fig. 3/Y"'). At 48 hpf apoptosis was also initiated in the smaller, vacuolated NC cells (Fig. 3/X, inset; yellow arrows in Fig. 6/F). Apoptosis in the NC remained restricted to these cells.

### U0126 treatment disrupts the chordamesoderm to mature notochord transition and proper signaling to surrounding tissues

**Figure 4:**
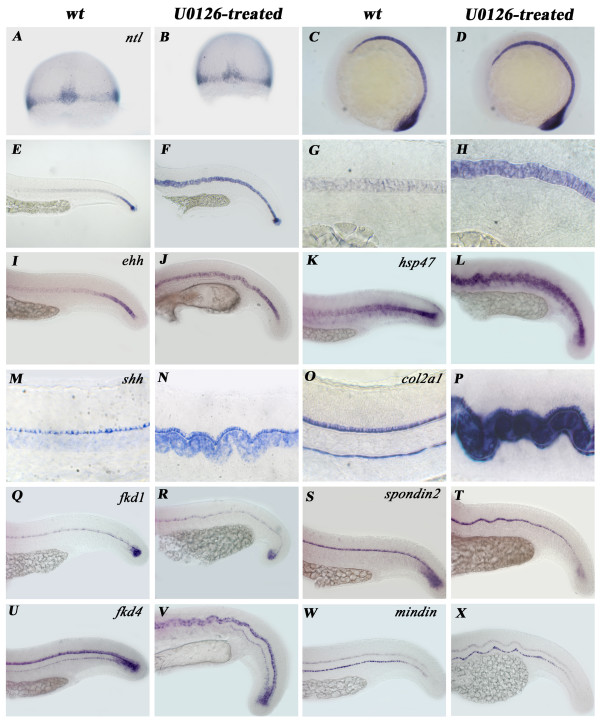
**Genes important in notochord differentiation exhibit two types of regulatory pattern during chordamesoderm to mature NC transition**. **A-H**: Expression of *ntl *is normal during early NC development (**A-D**) in treated embryos but fails to get downregulated during the chordamesoderm to mature NC transition step (**E-H**). (**A**, **B**: 7 hpf; **C**, **D**: 12 hpf; **E-H **24 hpf.) This pattern of developmental arrest occurs in *ehh *(**I**, **J**), *shh *(**L**, **K**), *col2α1 *(**M**, **N**) and *hsp47 *(**O**, **P**) expression. **Q-X**: mRNA levels of *fkd1 *(**Q**, **R**), *fkd4 *(**S**, **T**), *spondin2 *(**U**, **V**) and *mindin *(**W**, **X**) however decreases normally upon chordamesoderm to mature NC transition. Notice the unchanged expression of marker genes in the floor plate (**M-X**) and in the hypochord (**O**, **P**, **U-X**) of U0126-treated embryos. Panels: **A**, **C**, **E**, **G**, **I**, **K**, **M**, **O**, **Q**, **S**, **U**, **W**: wild type. Panels: **B**, **D**, **F**, **H**, **J**, **L**, **N**, **P**, **R**, **T**, **V**, **X**: U0126-treated embryos.

**Figure 5:**
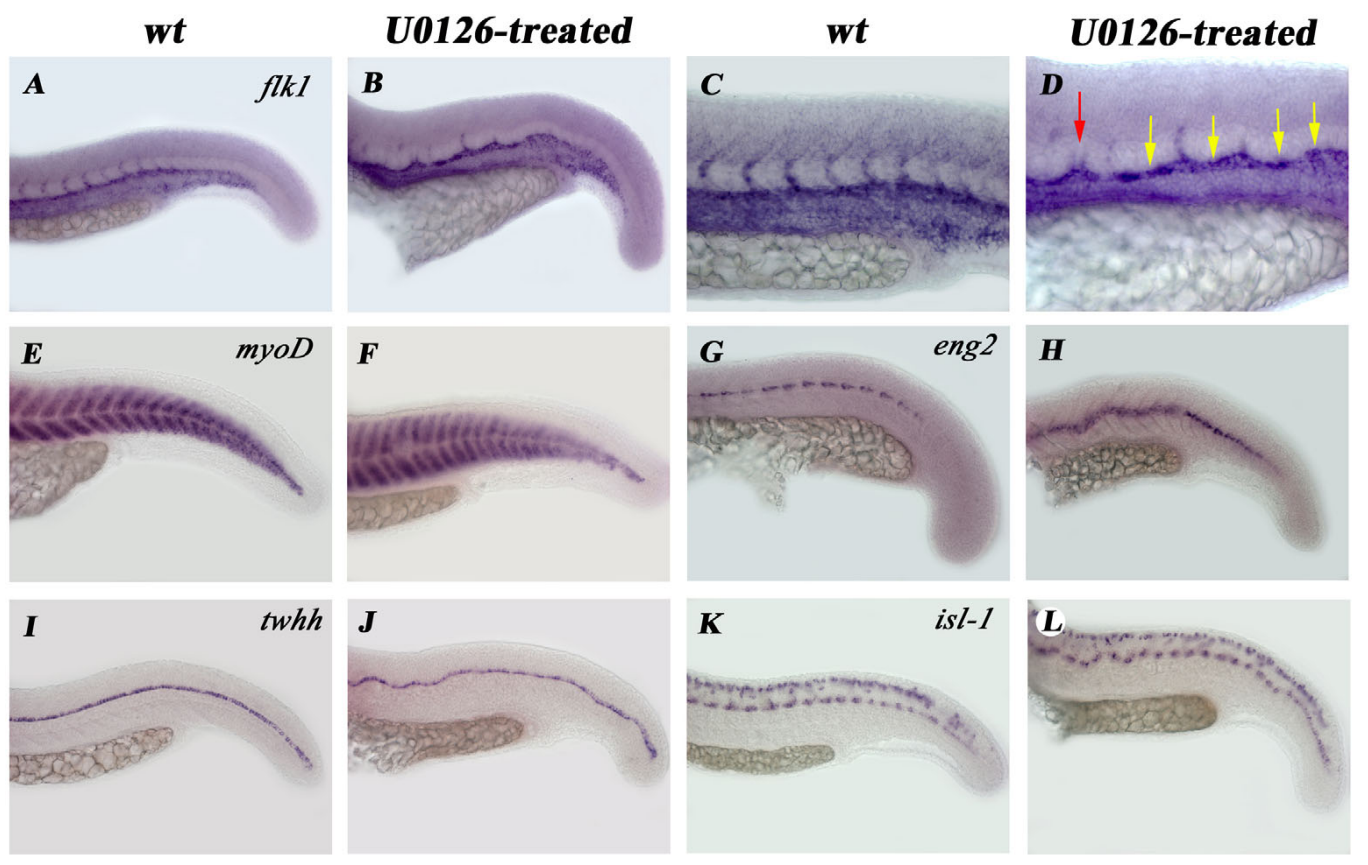
**Not all tissues receiving notochord-emitted signals are affected by U0126 treatment**. **A-D**: *flk-1 *expression reveals defects in dorsal aorta differentiation (green arrow in panel D) and in the sprouting of intersomitic vessels. Notice that both lack of sprouting (yellow arrows) and double sprouting (red arrow) occurs in U0126-treated embryos (**D**). Expression of the early muscle differentiation marker *myoD *is normal in treated embryos (**E**, **F**) but the muscle pioneer cell marker *eng2 *is upregulated (**G**, **H**). The floor plate of the neural tube differentiates normally (**I**, **J **and also **Fig. 4/M–X**). *Isl-1 *expression shows that the sensory Rohon-Beard neurons and motoneurons both develop properly in treated embryos (**K**, **L**)

To find the possible molecular consequences of U0126 treatment which led to the NC phenotype, we inspected the expression pattern of structural and regulatory genes involved in NC differentiation, most of which also play a role in tail development. *ntl*, the zebrafish orthologue of mouse Brachyury, is involved in NC and tail development in all vertebrates examined so far [46-50]. In wild type embryos, *ntl *is expressed in the NC during its development and from 20 hpf its expression progressively decays in an A-P fashion. Early expression of *ntl *was absolutely normal in U0126-treated zebrafish embryos (Fig. 4/A–D). From 20 hpf onwards however, unlike in untreated siblings, its expression did not decrease, in fact it was maintained at high levels (Fig. 4/E–H). This lack of proper mRNA decay in treated embryos also characterized expression of several other genes important in NC development and tail formation including *ehh *and *shh *(but not *twhh*) from the Hh family of signaling molecules, the structural protein *col2α1 *and its specific chaperone *hsp47 *(Fig. 4/I–P). In contrast, other genes also involved in NC development, such as members of type I forkhead transcription factors (*fkd1, fkd4*; Fig. 4/Q,R,U,V) as well as *tiggy-winkle hedgehog *(*twhh*; Fig. 5/I,J), *spondin2 *and *mindin*, showed normal expression dynamics in treated embryos (Fig. 4/Q–X).

Since expression of many genes belonging to developmentally important signaling pathways were affected by U0126-treatment, we examined whether differentiation of surrounding tissues known to require NC-emitted signals for their development (muscle, neural tube, circulatory system) were affected. *flk *and *tie-2 *expression, as well as *Fli1-eGFP *fluorescence all revealed considerable defects in circulatory system differentiation (Fig. 5/A–D; data not shown). Disruptions of *flk *gene expression were observed in the dorsal aorta (Fig. 5/D), and we also detected double sprouting of intersomitic vessel progenitors within the same somite (Fig. 5/D, red arrow). In most cases, however, there was no detectable sprouting at all from the precursors, suggesting that development of these vessels requires NC signals (Fig. 5/D, yellow arrows).

Early muscle differentiation markers showed only very mild or no defects (eg. *myoD *Fig. 5/E,F). Also, expression of the sarcomeric gene α-*tropomyosin *was not affected at all (data not shown). The level of expression of the slow muscle pioneer cell marker *eng2*, however, was significantly increased. Instead of its normal staining pattern, where only the anteriormost 2–3 cells in each somite at the somitic midline expresses *eng2 *mRNA, in U0126-treated embryos its expression spanned the entire myotome producing a continuous band along the D-V midline (Fig. 5/G,H).

**Figure 6:**
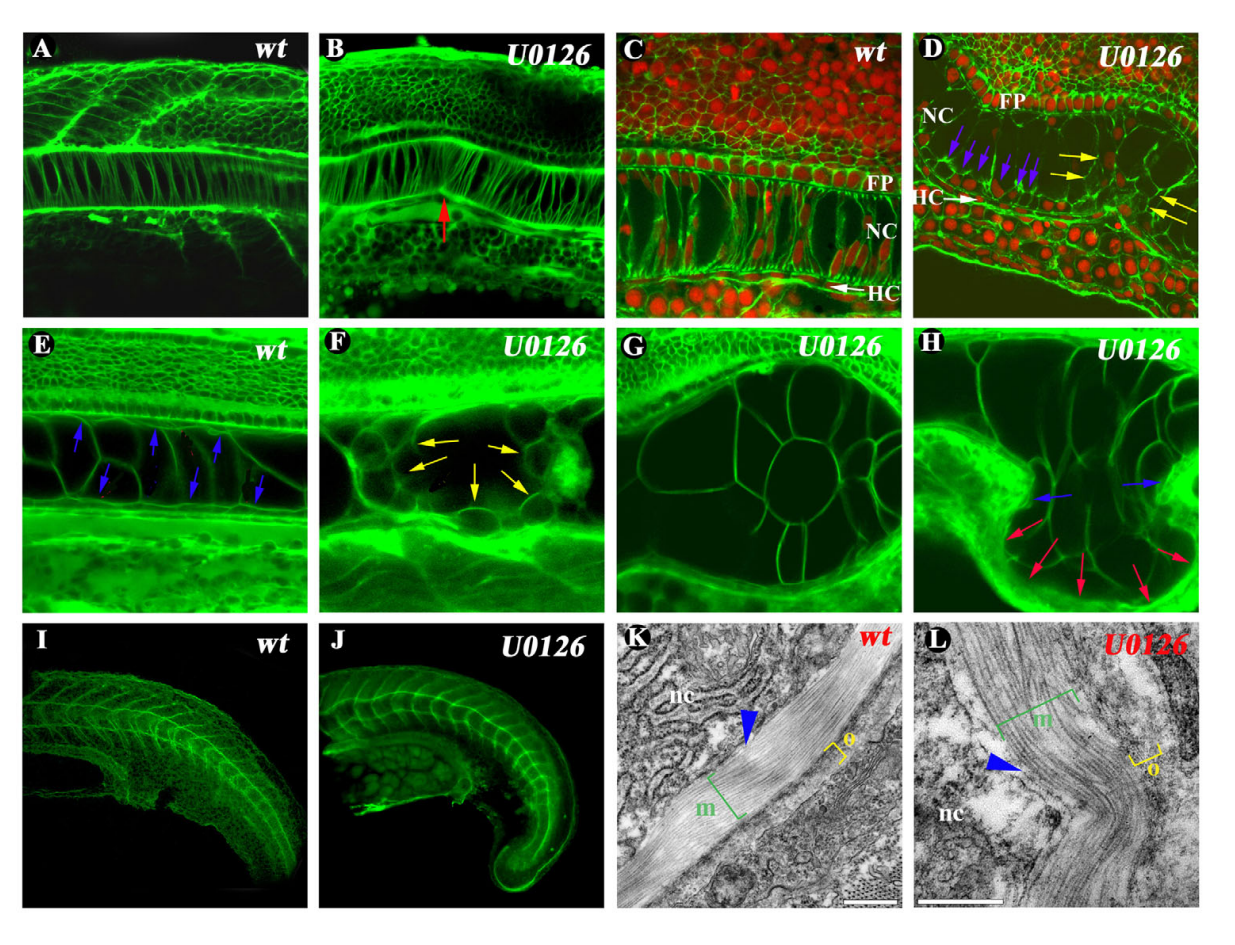
**Defects in the cellular organization of the notochord and PNBM structure of U0126-treated embryos**. In 24 hpf. U0126-treated embryos notochordal cells adhere to the sheath at preferential focal points which results in the bending of the NC (red arrow in panel **B**). Phalloidin stainings reveal smaller amount of fibrillar actin present between NC cells but not in the overlying neural tube. Notice the large vacuolar cells and the periferial positioning of nuclei in U0126-treated embryos (panels **C**, **D**). By 48 hpf. the NC of U0126-treated embryos with milder phenotypes contains smaller rounded cells which become apoptotic (yellow arrows in panel **F **and see also in **Fig. 3/X**). Embryos with stronger phenotypes have large bulges protruding where the flat sheath cells surrounding the large vacuolated cells are missing (see blue arrows in panel **E **for normal sheath cells and in panel **H **marking the last sheath cells surrounding the bulge; red arrows mark the region devoid of sheath cells). Laminin expression and ultrastructure of the inner layer of the PNBM (marked with blue arrowheads) composed mainly of laminins are normal in NC treated embryos as demonstrated by whole-mount immunhistochemistry (**I**, **J**) and TEM pictures (**K**, **L**). The middle (green bracket) and outer (yellow bracket) layers of the PNBM composed mainly of circumferential and longitudinal collagen fibers respectively show decreased length and/or disorganization in the orientation of fibers (**K**, **L**). Panels **A**, **C**, **E**, **G**, **I**, **K**: wild type embryos. Panels **B**, **D**, **F**, **H**, **J**, **L**: U0126-treated embryos.

The neural tube lies just dorsal to the NC and proper differentiation of its ventral part (floor plate, motoneurons) is dependent on notochordal signals. Despite this, and much to our surprise, the floor plate developed normally in U0126-treated embryos as judged both by its morphology (Fig. 6/A–D) and by the normal expression of *shh, twhh, col2α1, fkd1, fkd,4, spondin2 *and *mindin *genes (Fig. 5/I,J and Fig. 4/M,N,Q–X). Moreover, ventral motoneurons marked by *isl-1 *also differentiated properly (both in numbers as well as in position) from neural progenitors (Fig. 5/K,L).

### U0126 treatment induces changes in the actin based cytoskeleton and in the structure of the perinotochordal basement membrane

To investigate the NC morphology of treated embryos in more detail, we used the lipophilic vital dye Bodipy ceramide, which remains extracellular and outlines the borders of living cells. Staining of 24 hpf control embryos revealed a single row of cells in the NC arranged in a „stack-of-coins" shape (Fig. 6/A). At this age approximately every fourth or fifth cell already showed signs of vacuolation, which is the first visible morphological change in the chordamesoderm to NC differentiation process. In wild type embryos the NC cells were also still attached to the PNBM very evenly. In the NC of U0126-treated embryos this even distribution of cells was broken at several places, with 4–5 NC cells adhering to the PNBM at the same spot (See red arrow in Fig. 6/B). Such focused adhesion probably creates mechanical tension causing the NC to bend towards the adhesion point and might become an initiator during subsequent kinking/bending of the NC. The appearance of these structures suggested that there might be cell adhesion or/and cytoskeletal defects involved in the generation of the phenotype, so we next examined the distribution of fibrillar actin (F-actin) via fluorescent phalloidin staining (green in Fig. 6/C,D), with the nuclei being counterstained by Sytox dye (red).

In the medial part of the NC of U0126-treated embryos the phalloidin staining appeared significantly weaker indicating that a lower level of F-actin was present (Fig. 6/D). There was also a change in the appearance and rearrangement of notochordal cells with smaller cuboidal cells lying ventrally along the NC sheath (Fig. 6/D, blue arrows). Due to this, the large vacuolated cells were not connected to the sheath directly on the ventral side. At the points of NC breaks (or severe bends) these smaller cells intruded into medial parts of the NC (see yellow arrows in Fig. 6/D).

At 48 hpf the wild type NC contained large vacuolated cells with 2–3 similar size cells lying side by side (Fig. 6/E), and smaller flat epithelial-like sheath cells towards the edge, in close contact with the PNBM (Fig. 6/E, blue arrows). In U0126-treated embryos with a milder phenotype a large number of significantly smaller rounded cells with a vacuolated appearance were observed (Fig. 6/F, yellow arrows). It is not clear whether these cells are formed from the flat sheet cells secondarily or whether they are normal vacuolated cells that are simply smaller. In more severely affected U0126-treated embryos (Fig. 6/G,H), the flat sheath cells were not present continously along the NC and where they were missing, large bulges appeared in the NC consisting of large vacuolated cells (last sheath cells are marked with blue arrows on both sides of the bulge in the NC in Fig. 6/H). It is likely that the sheath cells, together with the PNBM, keep the normal shape of the NC by resisting the high internal hydrostatic pressure of vacuolated cells. The absence of the sheath cells in U0126 treated embryos may account for the formation of the large bulges in the NC.

As the observed phenotype resembles zebrafish laminin mutants [51,52], we next checked the presence and distribution of laminin protein around the NC in U0126-treated embryos by whole-mount immunhistochemistry (Fig. 6/I–J.). Laminin is expressed normally in treated embryos, suggesting that U0126 affects events occuring downstream or in parallel with laminin in NC differentiation. This was also supported by ultrastructural evidence from transmission electron microscopic (TEM) sections which showed that in 48 hpf embryos the laminin-based inner layer of the PNBM was not affected by U0126 treatment (Fig. 6/K,L, blue arrowheads). The medial (green bracket) and outer layers (yellow bracket) composed mostly of longitudinally and perpendicularly oriented crosslinked collagen fibres (mainly type IV) however showed some disorganization in U0126-treated embryos (Fig. 6/K,L). This was particularly evident in areas where the profile of the NC was 'kinked'. In these regions of the sheath the inner layer of collagen filaments was thinner and contained shorter collagen fibrils. These 'shorter' fibrils may be truly shorter or may appear shorter in the ultrathin sections because their orientation is more longitudinal rather than circumferential to the NC. Either way, these observations suggest some disorganization in the collagen layers.

In summary, we can conclude that most probably a combination of the lack of sheath cells and the disorganiation of the inner layer of the PNBM causes the multiple-cell wide bulgings in the NC of U0126-treated embryos. Although it is likely, at this point we can not yet conclude, whether there is a direct causal relationship between the missing sheath cells and the PNBM disorganization.

### U0126-treated embryos do not resemble FGF pathway-inhibited embryos

**Figure 7:**
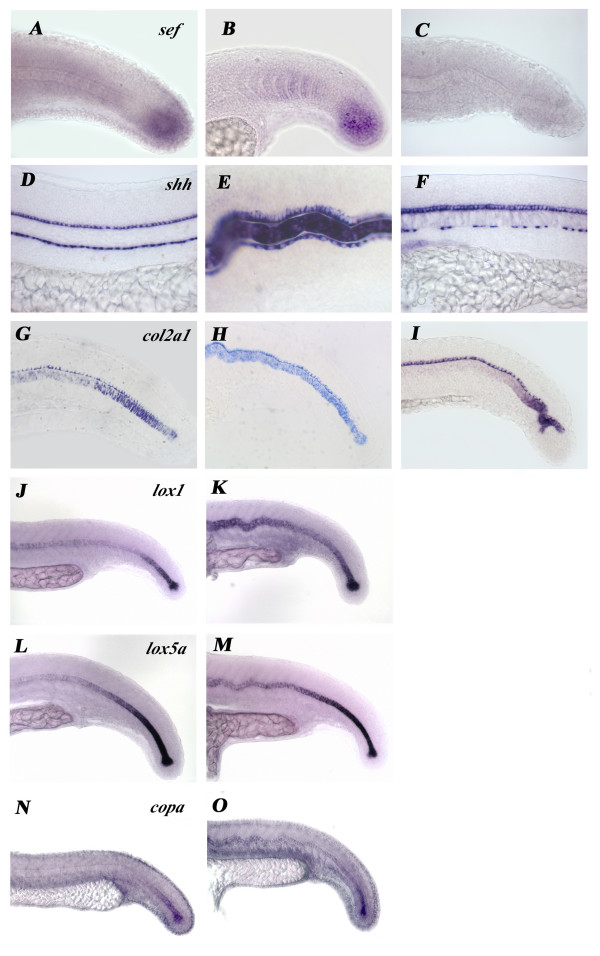
**Inhibition of the FGF pathway by SU5402 does not show any similarities to U0126-treated embryos**. **A-C**: Expression of the Fgf-pathway target gene *sef *is downregulated in SU5402 (**C**) but not in U0126-treated (**B**) embryos. **D-I**: Expression of the notochordal marker genes *col2a1 *(**D-F**) and *shh *(**G-I**) also show different defects in SU5402 and U0126-treated embryos. **J-M**: Expression of *lox1 *(**J**, **K**) and *lox5b *(**L-M**) is not decreased normally by U0126-treatment. **N**, **O**: *copa *mRNA persists and is not downregulated by U0126-treatment.

As the FGF pathway is known to transmit its signals via the MAPK-Erk pathway and it is also involved in trunk and tail development, we examined whether U0126-treated embryos resemble those in which the FGF-pathway was abrogated. We used the FGF pathway inhibitor SU5402, which has previously been shown to inhibit the FGF pathway *in vivo *in zebrafish embryos [38,53]. However, phenotypes of embryos treated with SU5402 in the same time window as U0126lacked any similarity to U0126-treated embryos. Also, the Fgf-target gene *sef *(as well as *pea3 *and *sprouty4*; data not shown), which was downregulated in the somites and tail mesenchyme of SU5402-treated embryos, was not affected by U0126 treatment (Fig. 7/A–C). Moreover, the notochordal markers *col2a1 *and *shh *maintained a normal A-P pattern of expression decay in the NC of SU5402-treated embryos (Fig. 7/D–I). These data suggest that FGF-signaling is unlikely to be involved in the activation of MEK in the context of NC differentiation.

The disorganisation found in the NC sheath of U0126-treated embryos is also a characteristic of zebrafish mutants which affect COPI-mediated secretion processes [54] or those with inhibiton of proper copper metabolism via the copper-transporter Atp7ase [55]. Therefore we investigated whether expression of the available coatomer complex and copper-dependent lysyl-oxidase genes are changed in U0126-treated embryos. The mRNA levels of some of these genes (*lox1, copa*) were abnormally maintained at high levels in treated embryos (Fig. 7/J,K,N,O) or decayed normally in other cases (lox5a; Fig. 7/L,M) similarly to what was observed for other notochordal genes. This suggests that there is no direct regulation of these genes by the ERK pathway at the transcriptional level but it is still possible that such a regulatory interaction exists at the protein level. These studies will have to wait until specific antibodies are available for all phosphorylated and unphosphorylated COPI and lysyl-oxidase isoforms.

## Discussion

### U0126 produces a specific effect during NC development in zebrafish

As a welcome side-effect of large scale small molecule screens, developmental biologists now have access to specific inhibitors of various signaling pathways which could provide a simple solution for a temporally controllable inhibition of developmentally crucial pathways.

MEK1 and MEK2 underscore the usefulness of the chemical genetics approach. *Mek1-/- *mouse embryos die at E10.5 due to defective development of placental circulation [56], while *Mek2-/- *animals are viable, fertile and have no visible phenotype [57]. To reveal possible later and/or redundant functions of these kinases, we applied their inhibitor U0126 to live zebrafish embryos. Our results demonstrate a potential new role for the the MAPK pathway in NC differentiation.

The specificity of U0126 has been previously demonstrated in zebrafish *in vivo *by Peterson and coworkers [33]. Here we show that U0126 effectively inhibits Erk1/2 phosphorylation in zebrafish embryos when applied from early gastrulation, and this correlates with the NC defect detected in our experiments. Treatment of embryos with U0124, a small molecule similar to U0126 but with a slightly changed composition, which results in loss of MEK1/2 inhibitory capacity, did not lead to NC defects. Accordingly, Erk1/2 phosphorylation was also unaffected by treatment with this drug. These results suggest that U0126 has a specific effect during NC differentiation. We tried a second inhibitor of MEK1/2, PD98059, which is 100-fold less potent than U0126. Unfortunately, PD98059 treatments were toxic to the embryos at 30 μM, and at sublethal concentrations (20–25μM) the embryos did not show any NC phenotype nor any decrease in Erk1/2 phosphorylation. Given the *in vitro *data and our results we have to conclude that the toxicity of this drug did not allow us to to compare its effect on NC development with that of U0126.

### U0126 disrupts the chordamesoderm to mature NC transition process

Our data show that U0126 does not affect early patterning and morphogenetic events; rather it disrupts a later step during NC differentiation. This is in contrast to Ascidians where application of U0126 disrupts an early NC determination step. In Ciona intestinalis it also decreases expression of the early NC determination gene *Ci-Bra*, the ascidian orthologue of zebrafish *ntl *which as we demonstrated does not show any expression defects during early stages of NC differentiation [29,58]. This suggests that the U0126 target protein has an earlier function in Ascidian NC morphogenesis then in zebrafish. Alternatively, the drug may penetrate more rapidly into the significantly smaller Ascidian embryos.

In zebrafish, at around 20 hpf chordamesoderm cells start to differentiate into mature NC and the mRNA levels of genes required for the early development and signaling function of the chordamesoderm are downregulated. Simultaneously, the cells of the differentiating NC begin to secrete the components of the PNBM, a structure which will support the NC against its own hydrostatic pressure and maintain its classic rod-like structure. Our expression analysis revealed that mRNA levels of important regulatory and structural genes are not downregulated in U0126-treated embryos indicating an arrest of NC differentiation at a stage which approximately corresponds to a 16 hpf embryo. Surprisingly, despite this, the surrounding tissues are only mildly (or not at all) affected with the exception of the developing circulatory system, which was analyzed in detail recently by Peterson and colleagues; [33], and the slow muscle pioneer cells, which are increased in number and expand in an A-P direction within the somitic midline The ventral neural tube (floor plate and the motoneurons, which also both require proper Hh-signaling from the NC however, differentiate properly. This mixed phenotype suggests that some tissues are not affected because the arrest in NC development happens only after it has already provided its essential signals to them (eg. to the neural tube), while others would still require proper signaling from the NC (eg. circulatory system, slow muscle pioneer cells). Alternatively, the difference is due to ability of the surrounding tissues to cope with the abnormal signals they recieve. Finally, it is possible that the NC cells are capable of handling the extra mRNA by translational or posttranslational regulatory mechanisms. This is supported by the fact that GFP protein levels in the NC of U0126-treated *shh-GFP *embryos appear to be downregulated normally by 48 hpf [data not shown; [45,59]].

Even though the effects of the U0126 treatment are not visible until late in somitogenesis, our time-course and washout experiments revealed that the drug has to be applied from early gastrulation to achieve a fully penetrant phenotype. In addition, the chemical has to be kept on continously; when treatments start at later stages only the posterior part of the NC is affected and conversely, early washout of the chemical produces undulations only in the more anterior part of the NC. This suggests that there is a sensitivity window to MEK activity that moves from anterior to posterior as the NC cells differentiate.

### FGF signaling is unlikely to be involved in the NC differentiation pathway targeted by U0126

We examined FGFs as candidates for the upstream signal in the U0126-targeted NC differentiation process. FGFs are known to transmit their signals via the MAPK pathway [37] and have been implicated in the tail elongation process [60]. Unfortunately, they are also involved in D-V patterning, which begins prior to NC differentiation [39]. As a result of this, *in vivo *disruption of FGF signaling, using SU5402, or by using a combination of *fgf3 *and *fgf8 *antisense morpholinos (our unpublished data), produced a severe phenotype which probably masks its later function during NC differentiation. The *fgf8 *mutant *ace *also does not have an undulating NC, indicating it is not the upstream signal in this pathway [61]. However, since U0126 treatments seem to be able to eliminate Erk1/2 phosphorylation, and FGF signalling is transduced by the activity of these kinases, it seems puzzling that it only generates a mild phenotype compared to that expected from a complete lack of FGF signalling. We are aware of this contradiction and currently do not have a proper answer for it. The penetration of U0126 (5 hours after application there was still pErk1/2 present as detected by our Western blot analyses) could potentially also contribute the lack of early FGF-inhibition-like phenotype.

It may also be possible that U0126 preferentially inhibits only Mek1 or Mek2, a hypothesis that cannot be tested due to lack of specific antibodies. Alternatively, some aspect of FGF signaling may be transduced by the activity of other molecules in addition to Mek1/2, and those parallel pathways are not inhibited by U0126.

### U0126 might inhibit the regulatory processes involved in the secretion of PNBM components

U0126 treatment phenocopies 3 groups of mutants identified in earlier large-scale mutagenesis screens [62,63]: the *laminin*, the *coatomer complex *(COPI) and the copper transporter *ATP7a *mutants [55,64,65]. They all have a shortened body axis and an undulating NC in which the chordamesoderm to mature NC transition process is arrested with similar abnormal persistence of mRNAs of structural and regulatory genes. Moreover, the structure of the PNBM is also perturbed in these mutants.

*laminin *mutants (*sleepy, grumpy and bashful*), however, differ from U0126-treated embryos in several features. The inner layer of the PNBM, consisting mostly of laminins [51,52], is severely perturbed in these mutants, while in U0126 treated embryos laminin distribution and ultrastructure of the inner layer of the PNBM is entirely normal. *laminin *mutants also have neural defects, while neural development is normal in U0126 treated embryos. In addition, U0126 treatments lead to other defects not present in these mutants, such as the lack of pigmentation and the presence of apoptosis between 48 and 72 hpf. These results suggest that the role of Mek1/2 signaling in NC differentiation is downstream of, or parallel to laminin.

The COPI mutants (*dopey, sneezy *and *happy *[54]) and the copper metabolism disrupting *calamity *mutant [55] very closely resemble U0126-treated embryos with no additional or missing features. COPI is an essential part of the mammalian secretory machinery [66,67] which participates in retrograde transport from Golgi to the ER. The COPI complex comprised of 7 subunits and detailed mechanism of its action during this process is still not clear. It is very interesting to note however that according to a recent paper, recruitment of the other coatomer complex, COPII to ER export sites is regulated by phosphorylation via the p38/MAPK-pathway [68]. Due to the lack of specific antibodies for phosphorylated and dephosphorylated versions of the seven COPI subunits we could not examine the possibility that Mek1/2 phosphorylation is involved in regulation of this pathway but it is a promising direction for future research.

### Interference with copper homeostasis phenocopies U0126-treatment in zebrafish embryos

The *calamity *zebrafish mutant (which bears a mutation on the copper transporter gene *ATP7a *and can also be phenocopied by copper chelating agents) [55,69] has a phenotype which is almost identical to that produced by U0126 treatment. Copper is an essential cofactor for the function of a variety of enzymes involved in iron oxidation, peptide amidation, cellular respiration and antioxidant defense [70]. The copper transporter Atp7a is also affected in Menkes disease in humans, which has skeletal defects among its symptoms [71].

We can exclude the possibility of U0126 acting simply as a copper chelator, since pretreatment of U0126 solution with CuCl_2 _or CuSO_4 _did not prevent it from inducing NC defects in the embryos (not shown). Still, our TEM results demonstrate strong similarities between U0126-treated embryos and embryos in which copper metabolism is interfered with. These similarities include a milder decrease in collagen mass and disorganized, shorter fibers in the PNBM. This suggests that the copper metabolism pathway or at least part of it is regulated by the U0126 target process, although the nature of this interaction is unclear at present. Lysyl-oxidase is a copper dependent amin-oxidase normally expressed in the NC, and involved in the posttranslational, extracellular crosslinking of collagens and elastin [72,73]. This enzyme is regulated by FGF and Ras via the MAPK pathway and copper chelation results in its inactivation. [74,75]. Recently, it has also been shown that lysyl-oxidase activity regulates actin-filament formation, a process also affected in the NC of U0126-treated embryos [76]. Thus, the U0126 treatment may inferfere with these processes directly or indirectly via lysyl-oxidase activity and further studies will be necessary to address this question.

## Conclusion

In summary, using pharmacological inhibition of Mek1/2 we demonstrated that:

i. U0126 treatment causes a consistent phenotype including lack of pigmentation and undulating NC which correlates with lack of Erk1/2 phosphorylation in zebrafish embryos.

ii. U0126 disrupts the NC differentiation process during the chordamesoderm to mature NC transition step. As a result, structural and signaling genes fail to be downregulated during this process.

iii. U0126-treatment also leads to abnormal differentiation and weakening of the PNBM. This leads to an inability of the PNBM to support the NC against its own hydrostatic pressure and results in undulations and bulges in its structure.

iv. Using various methods of in vivo inhibition we excluded FGF as the potential upstream signal in the NC differentiation process affected by U0126.

There are 2 major questions arising from our experiments:

1. Is it possible that despite the overwhelming previously published evidence proving its specificity (and our ERK phosphorylation results) U0126 also might affect more than just MEK1/2?

2. Is there a direct regulatory interaction between the COPI-mediated retrograde transport, the copper-dependent postranslational modifications by lysyl-oxidase and the U0126-targeted pathway?

The answer to these questions might surface through the cloning and identification of further uncharacterized mutants with similar phenotypes (ie. *crash test dummy, leviathan*). Alternatively, the identification of further pathway-specific chemicals in large-scale pharmacological screens which cause similar developmental defects could help in elucidating this phenomenon.

## Methods

### Fish maintainance and MEK1/2-inhibitor treatments

Zebrafish (Danio rerio) were maintained under standard conditions [77]. Embryos were collected from wild-type (wik strain) crossings and kept at 28.5 C. Developmental stages were identified based on [78]. U0126 (Promega, V1121) was dissolved according to suppliers information (10 mM in DMSO). Up to 50 embryos were treated in 12-well plates with the appropriate concentration of U0126 diluted from stock in fish water. Embryos were kept in the solution until fixation. 10 mM stock solutions of U0124 (Santa Cruz Biochemicals) and PD98059 (Calbiochem) were made using DMSO. Embryos were treated as per U0126 with the appropriate concentrations of the drug (100 μM for U0124; 20, 25 and 30 μM for PD98059). U0124 was dissolved in 2% DMSO fish water as it precipitated otherwise, the appropriate control was used to ensure no effect of the high DMSO treatment.

### In situ hybridizations, TUNEL stainings

Probe synthesis and whole mount in situ hybridizations (WISH) were carried out as described previously [79] using DIG labeling kit and NBT/BCIP developing solution from LaRoche. Two-color WISH was done using DIG and Fluorescein labelled probes recognized by the proper Fab fragments linked to alkaline phosphatase enzymes. The first probe was developed with NBT (La Roche) then fixed, washed in PBST (PBS w. 0.1% Tween20), the antibody destroyed in 0.2 M glycine solution washed again with PBST, blocked and incubated with the anti-Fluorescein-AP linked Fab fragment. After extensive washing this Ab was developed using INT/BCIP solution (La Roche).

TUNEL stainings were carried out according to manufacturer's recommendation (Apoptaq kit, Chemicon)

### Immunhistochemistry, Bodipy-ceramide, phalloidin-sytox stainings and microscopy

Standard procedures of whole mount immunhistochemistry were carried out using α-Acetylated tubulin Ab from (Sigma; 1:1000), α-phospho-histone3 Ab (Upstate; 1:400) and α-laminin (Sigma; 1:400) α-pERK1/2 (Sigma; 1:250). In vivo Bodipy-ceramide stainings were done as previously described [80]. Alexa568-conjugated phalloidin and Sytox-green (both Molecular Probes) stainings were carried out on PFA-fixed embryos according to manufacturers' protocols. All stained embryos were mounted on a slide with a coverslip and photographed on a Leica TCS2 confocal microscope.

For electron microscopy embryos were fixed in 2% paraformaldehyde, 2% gluteraldehyde in 0.1 M sodium cacodylate buffer (pH 7.3) with 2% CaCl_2_. Following postfixation in cacodylate buffered 1% w/v osmium tetraoxide (OsO_4_) (Johnson-Matthey) tissue was *en bloc *stained in 2% w/v uranyl acetate and embedded in medium Agar 100 epoxy resin (Agar Scientific). Ultrathin sections (80–90 nm) contrasted with saturated uranyl acetate solution and lead-citrate were examined and photographed using a JEOL1010 electron microscope.

### SDS-PAGE/Western Blotting

Protein was extracted by homogenisation of deyolked embryos, 7.5 μg of protein was seperated using 10% polyacrylamide gels, followed by transfer onto PVDF membrane (Amersham). The pERK1/2 antibody was the same as for immunohistochemistry applied here at 1:5000, mouse anti-gamma tubulin (Sigma; 1:10,000) was the loading control. Peroxidase activity from HRP-conjugated secondaries (Sigma) was detected using an ECL detection kit (Amersham).

## List of Abbreviations

Ab: antibody; C-E: convergent-extension; COPI: coatomer complex I; DIG: digoxygenin; ER: endoplasmatic reticulum; ERK: Extracellular signal regulated kinase; FP: floor plate; hh: hedgehog; hpf: hours post.fertilization; HC: hypochord; MAPK: Mitogen activated protein kinase; MEK: MAPK/ERK kinase; NC: notochord; PBS: phosphate buffered saline; PFA: parafolmaldehyde; PNBM: perinotochordal basement membrane; TEM: transmission eletron microscope; TUNEL: Terminal deoxynucleotidyl transferase dUTP Nick End Labeling; WISH: whole-mount in situ hybridization; wt: wild type; Zf: zebrafish.

## Authors' contributions

TH did in situ hybridizations as well as electron microscopy and was involved in writing the manuscript. FC did the TUNEL stainings and in situ hybridizations and was involved in writing the manuscript. FE did U0126 inhibitor experiments, GS was involved in planning the experiments and writing the manuscript. ZL concieved the project, carried out large part of the experiments and wrote the manuscript.

## References

[B1] Lele Z, Krone PH (1996). The zebrafish as a model system in developmental, toxicological and transgenic research. Biotechnology Advances.

[B2] Teraoka H, Russell C, Regan J, Chandrasekhar A, Concha ML, Yokoyama R, Higashi K, Take-Uchi M, Dong W, Hiraga T, Holder N, Wilson SW (2004). Hedgehog and Fgf signaling pathways regulate the development of tphR-expressing serotonergic raphe neurons in zebrafish embryos. J Neurobiol.

[B3] Chen W, Burgess S, Hopkins N (2001). Analysis of the zebrafish smoothened mutant reveals conserved and divergent functions of hedgehog activity. Development.

[B4] Griffin KJ, Kimelman D (2003). Interplay between FGF, one-eyed pinhead, and T-box transcription factors during zebrafish posterior development. Dev Biol.

[B5] Gunhaga L, Marklund M, Sjodal M, Hsieh JC, Jessell TM, Edlund T (2003). Specification of dorsal telencephalic character by sequential Wnt and FGF signaling. Nat Neurosci.

[B6] Hagos EG, Dougan ST (2007). Time-dependent patterning of the mesoderm and endoderm by Nodal signals in zebrafish. BMC Dev Biol.

[B7] Peterson JR, Lebensohn AM, Pelish HE, Kirschner MW (2006). Biochemical suppression of small-molecule inhibitors: a strategy to identify inhibitor targets and signaling pathway components. Chem Biol.

[B8] Zon LI, Peterson RT (2005). In vivo drug discovery in the zebrafish. Nature reviews.

[B9] Favata MF, Horiuchi KY, Manos EJ, Daulerio AJ, Stradley DA, Feeser WS, Van Dyk DE, Pitts WJ, Earl RA, Hobbs F, Copeland RA, Magolda RL, Scherle PA, Trzaskos JM (1998). Identification of a novel inhibitor of mitogen-activated protein kinase kinase. J Biol Chem.

[B10] Garrington TP, Johnson GL (1999). Organization and regulation of mitogen-activated protein kinase signaling pathways. Curr Opin Cell Biol.

[B11] Nebreda AR, Porras A (2000). p38 MAP kinases: beyond the stress response. Trends Biochem Sci.

[B12] Weston CR, Davis RJ (2002). The JNK signal transduction pathway. Curr Opin Genet Dev.

[B13] Roux PP, Blenis J (2004). ERK and p38 MAPK-activated protein kinases: a family of protein kinases with diverse biological functions. Microbiol Mol Biol Rev.

[B14] Krens SF, Spaink HP, Snaar-Jagalska BE (2006). Functions of the MAPK family in vertebrate-development. FEBS Lett.

[B15] Lloyd AC (2006). Distinct functions for ERKs?. J Biol.

[B16] Nishimoto S, Nishida E (2006). MAPK signalling: ERK5 versus ERK1/2. EMBO Rep.

[B17] Krens SF, He S, Spaink HP, Snaar-Jagalska BE (2006). Characterization and expression patterns of the MAPK family in zebrafish. Gene Expr Patterns.

[B18] Duncia JV, Santella JB, Higley CA, Pitts WJ, Wityak J, Frietze WE, Rankin FW, Sun JH, Earl RA, Tabaka AC, Teleha CA, Blom KF, Favata MF, Manos EJ, Daulerio AJ, Stradley DA, Horiuchi K, Copeland RA, Scherle PA, Trzaskos JM, Magolda RL, Trainor GL, Wexler RR, Hobbs FW, Olson RE (1998). MEK inhibitors: the chemistry and biological activity of U0126, its analogs, and cyclization products. Bioorg Med Chem Lett.

[B19] Giuliani R, Bastaki M, Coltrini D, Presta M (1999). Role of endothelial cell extracellular signal-regulated kinase1/2 in urokinase-type plasminogen activator upregulation and in vitro angiogenesis by fibroblast growth factor-2. J Cell Sci.

[B20] Hong HJ, Chan P, Liu JC, Juan SH, Huang MT, Lin JG, Cheng TH (2004). Angiotensin II induces endothelin-1 gene expression via extracellular signal-regulated kinase pathway in rat aortic smooth muscle cells. Cardiovasc Res.

[B21] Hida M, Omori S, Awazu M (2002). ERK and p38 MAP kinase are required for rat renal development. Kidney Int.

[B22] Hellman NE, Greco AJ, Rogers KK, Kanchagar C, Balkovetz DF, Lipschutz JH (2005). Activated extracellular signal-regulated kinases are necessary and sufficient to initiate tubulogenesis in renal tubular MDCK strain I cell cysts. Am J Physiol Renal Physiol.

[B23] Delfini MC, Dubrulle J, Malapert P, Chal J, Pourquie O (2005). Control of the segmentation process by graded MAPK/ERK activation in the chick embryo. Proc Natl Acad Sci U S A.

[B24] Zatechka SD, Lou MF (2002). Studies of the mitogen-activated protein kinases and phosphatidylinositol-3 kinase in the lens. 1. The mitogenic and stress responses. Exp Eye Res.

[B25] Webber CA, Chen YY, Hehr CL, Johnston J, McFarlane S (2005). Multiple signaling pathways regulate FGF-2-induced retinal ganglion cell neurite extension and growth cone guidance. Mol Cell Neurosci.

[B26] Naska S, Cenni MC, Menna E, Maffei L (2004). ERK signaling is required for eye-specific retino-geniculate segregation. Development.

[B27] Hudson C, Darras S, Caillol D, Yasuo H, Lemaire P (2003). A conserved role for the MEK signalling pathway in neural tissue specification and posteriorisation in the invertebrate chordate, the ascidian Ciona intestinalis. Development.

[B28] Kim GJ, Nishida H (2001). Role of the FGF and MEK signaling pathway in the ascidian embryo. Dev Growth Differ.

[B29] Minokawa T, Yagi K, Makabe KW, Nishida H (2001). Binary specification of nerve cord and notochord cell fates in ascidian embryos. Development.

[B30] Stemple DL (2005). Structure and function of the notochord: an essential organ for chordate development. Development.

[B31] Murakami S, Kan M, McKeehan WL, de Crombrugghe B (2000). Up-regulation of the chondrogenic Sox9 gene by fibroblast growth factors is mediated by the mitogen-activated protein kinase pathway
10.1073/pnas.97.3.1113. Proceedings of the National Academy of Sciences.

[B32] Bedogni B, O'Neill MS, Welford SM, Bouley DM, Giaccia AJ, Denko NC, Powell MB (2004). Topical Treatment with Inhibitors of the Phosphatidylinositol 3'-Kinase/Akt and Raf/Mitogen-Activated Protein Kinase Kinase/Extracellular Signal-Regulated Kinase Pathways Reduces Melanoma Development in Severe Combined Immunodeficient Mice
10.1158/0008-5472.CAN-03-3327. Cancer Res.

[B33] Hong CC, Peterson QP, Hong JY, Peterson RT (2006). Artery/vein specification is governed by opposing phosphatidylinositol-3 kinase and MAP kinase/ERK signaling. Curr Biol.

[B34] Dudley DT, Pang L, Decker SJ, Bridges AJ, Saltiel AR (1995). A Synthetic Inhibitor of the Mitogen-Activated Protein Kinase Cascade
10.1073/pnas.92.17.7686. Proceedings of the National Academy of Sciences.

[B35] Deacon SW, Nascimento A, Serpinskaya AS, Gelfand VI (2005). Regulation of bidirectional melanosome transport by organelle bound MAP kinase. Curr Biol.

[B36] Furthauer M, Thisse C, Thisse B (1997). A role for FGF-8 in the dorsoventral patterning of the zebrafish gastrula. Development.

[B37] Furthauer M, Reifers F, Brand M, Thisse B, Thisse C (2001). sprouty4 acts in vivo as a feedback-induced antagonist of FGF signaling in zebrafish. Development.

[B38] Furthauer M, Lin W, Ang SL, Thisse B, Thisse C (2002). Sef is a feedback-induced antagonist of Ras/MAPK-mediated FGF signalling. Nat Cell Biol.

[B39] Furthauer M, Van Celst J, Thisse C, Thisse B (2004). Fgf signalling controls the dorsoventral patterning of the zebrafish embryo. Development.

[B40] Hammerschmidt M, Serbedzija GN, McMahon AP (1996). Genetic analysis of dorsoventral pattern formation in the zebrafish: requirement of a BMP-like ventralizing activity and its dorsal repressor. Genes Dev.

[B41] Mullins MC, Hammerschmidt M, Kane DA, Odenthal J, Brand M, van Eeden FJ, Furutani-Seiki M, Granato M, Haffter P, Heisenberg CP, Jiang YJ, Kelsh RN, Nusslein-Volhard C (1996). Genes establishing dorsoventral pattern formation in the zebrafish embryo: the ventral specifying genes. Development.

[B42] Solnica-Krezel L, Stemple DL, Mountcastle-Shah E, Rangini Z, Neuhauss SC, Malicki J, Schier AF, Stainier DY, Zwartkruis F, Abdelilah S, Driever W (1996). Mutations affecting cell fates and cellular rearrangements during gastrulation in zebrafish. Development.

[B43] Marlow F, Zwartkruis F, Malicki J, Neuhauss SC, Abbas L, Weaver M, Driever W, Solnica-Krezel L (1998). Functional interactions of genes mediating convergent extension, knypek and trilobite, during the partitioning of the eye primordium in zebrafish. Dev Biol.

[B44] Topczewski J, Sepich DS, Myers DC, Walker C, Amores A, Lele Z, Hammerschmidt M, Postlethwait J, Solnica-Krezel L (2001). The zebrafish glypican knypek controls cell polarity during gastrulation movements of convergent extension. Dev Cell.

[B45] Shkumatava A, Fischer S, Muller F, Strahle U, Neumann CJ (2004). Sonic hedgehog, secreted by amacrine cells, acts as a short-range signal to direct differentiation and lamination in the zebrafish retina
10.1242/dev.01247. Development.

[B46] Schulte-Merker S, van Eeden FJ, Halpern ME, Kimmel CB, Nusslein-Volhard C (1994). no tail (ntl) is the zebrafish homologue of the mouse T (Brachyury) gene. Development.

[B47] Halpern ME, Ho RK, Walker C, Kimmel CB (1993). Induction of muscle pioneers and floor plate is distinguished by the zebrafish no tail mutation. Cell.

[B48] Herrmann BG (1992). Action of the Brachyury gene in mouse embryogenesis. Ciba Found Symp.

[B49] Wilkinson DG, Bhatt S, Herrmann BG (1990). Expression pattern of the mouse T gene and its role in mesoderm formation. Nature.

[B50] Kispert A, Herrmann BG (1994). Immunohistochemical analysis of the Brachyury protein in wild-type and mutant mouse embryos. Dev Biol.

[B51] Parsons MJ, Pollard SM, Saude L, Feldman B, Coutinho P, Hirst EM, Stemple DL (2002). Zebrafish mutants identify an essential role for laminins in notochord formation. Development.

[B52] Pollard SM, Parsons MJ, Kamei M, Kettleborough RN, Thomas KA, Pham VN, Bae MK, Scott A, Weinstein BM, Stemple DL (2006). Essential and overlapping roles for laminin alpha chains in notochord and blood vessel formation. Dev Biol.

[B53] Shinya M, Koshida S, Sawada A, Kuroiwa A, Takeda H (2001). Fgf signalling through MAPK cascade is required for development of the subpallial telencephalon in zebrafish embryos. Development.

[B54] Coutinho P, Parsons MJ, Thomas KA, Hirst EM, Saude L, Campos I, Williams PH, Stemple DL (2004). Differential requirements for COPI transport during vertebrate early development. Dev Cell.

[B55] Mendelsohn BA, Yin C, Johnson SL, Wilm TP, Solnica-Krezel L, Gitlin JD (2006). Atp7a determines a hierarchy of copper metabolism essential for notochord development. Cell Metab.

[B56] Giroux S, Tremblay M, Bernard D, Cardin-Girard JF, Aubry S, Larouche L, Rousseau S, Huot J, Landry J, Jeannotte L, Charron J (1999). Embryonic death of Mek1-deficient mice reveals a role for this kinase in angiogenesis in the labyrinthine region of the placenta. Curr Biol.

[B57] Belanger LF, Roy S, Tremblay M, Brott B, Steff AM, Mourad W, Hugo P, Erikson R, Charron J (2003). Mek2 is dispensable for mouse growth and development. Mol Cell Biol.

[B58] Sakabe E, Tanaka N, Shimozono N, Gojobori T, Fujiwara S (2006). Effects of U0126 and fibroblast growth factor on gene expression profile in Ciona intestinalis embryos as revealed by microarray analysis. Dev Growth Differ.

[B59] Hadzhiev Y, Lele Z, Schindler S, Wilson SW, Ahlberg P, Strahle U, Muller F (2007). Hedgehog signaling patterns the outgrowth of unpaired skeletal appendages in zebrafish. BMC Dev Biol.

[B60] Griffin K, Patient R, Holder N (1995). Analysis of FGF function in normal and no tail zebrafish embryos reveals separate mechanisms for formation of the trunk and the tail. Development.

[B61] Brand M, Heisenberg CP, Warga RM, Pelegri F, Karlstrom RO, Beuchle D, Picker A, Jiang YJ, Furutani-Seiki M, van Eeden FJ, Granato M, Haffter P, Hammerschmidt M, Kane DA, Kelsh RN, Mullins MC, Odenthal J, Nusslein-Volhard C (1996). Mutations affecting development of the midline and general body shape during zebrafish embryogenesis. Development.

[B62] Driever W, Solnica-Krezel L, Schier AF, Neuhauss SC, Malicki J, Stemple DL, Stainier DY, Zwartkruis F, Abdelilah S, Rangini Z, Belak J, Boggs C (1996). A genetic screen for mutations affecting embryogenesis in zebrafish. Development.

[B63] Haffter P, Granato M, Brand M, Mullins MC, Hammerschmidt M, Kane DA, Odenthal J, van Eeden FJ, Jiang YJ, Heisenberg CP, Kelsh RN, Furutani-Seiki M, Vogelsang E, Beuchle D, Schach U, Fabian C, Nusslein-Volhard C (1996). The identification of genes with unique and essential functions in the development of the zebrafish, Danio rerio. Development.

[B64] Odenthal J, Haffter P, Vogelsang E, Brand M, van Eeden FJ, Furutani-Seiki M, Granato M, Hammerschmidt M, Heisenberg CP, Jiang YJ, Kane DA, Kelsh RN, Mullins MC, Warga RM, Allende ML, Weinberg ES, Nusslein-Volhard C (1996). Mutations affecting the formation of the notochord in the zebrafish, Danio rerio. Development.

[B65] Stemple DL, Solnica-Krezel L, Zwartkruis F, Neuhauss SC, Schier AF, Malicki J, Stainier DY, Abdelilah S, Rangini Z, Mountcastle-Shah E, Driever W (1996). Mutations affecting development of the notochord in zebrafish. Development.

[B66] Bannykh SI, Nishimura N, Balch WE (1998). Getting into the Golgi. Trends Cell Biol.

[B67] Nickel W, Brugger B, Wieland FT (2002). Vesicular transport: the core machinery of COPI recruitment and budding. J Cell Sci.

[B68] Wang L, Lucocq JM (2007). p38 MAPK regulates COPII recruitment. Biochemical and Biophysical Research Communications.

[B69] Anderson C, Bartlett SJ, Gansner JM, Wilson D, He L, Gitlin JD, Kelsh RN, Dowden J (2007). Chemical genetics suggests a critical role for lysyl oxidase in zebrafish notochord morphogenesis. Mol Biosyst.

[B70] Pena MM, Lee J, Thiele DJ (1999). A delicate balance: homeostatic control of copper uptake and distribution. J Nutr.

[B71] Menkes JH (1999). Menkes disease and Wilson disease: two sides of the same copper coin. Part I: Menkes disease. Eur J Paediatr Neurol.

[B72] Molnar J, Fong KS, He QP, Hayashi K, Kim Y, Fong SF, Fogelgren B, Szauter KM, Mink M, Csiszar K (2003). Structural and functional diversity of lysyl oxidase and the LOX-like proteins. Biochim Biophys Acta.

[B73] Gansner JM, Mendelsohn BA, Hultman KA, Johnson SL, Gitlin JD (2007). Essential role of lysyl oxidases in notochord development. Developmental Biology.

[B74] Feres-Filho EJ, Menassa GB, Trackman PC (1996). Regulation of lysyl oxidase by basic fibroblast growth factor in osteoblastic MC3T3-E1 cells. J Biol Chem.

[B75] Contente S, Kenyon K, Sriraman P, Subramanyan S, Friedman RM (1999). Epigenetic inhibition of lysyl oxidase transcription after transformation by ras oncogene. Mol Cell Biochem.

[B76] Payne SL, Hendrix MJ, Kirschmann DA (2006). Lysyl oxidase regulates actin filament formation through the p130(Cas)/Crk/DOCK180 signaling complex. J Cell Biochem.

[B77] Westerfield M (1993). The zebrafish book. University of Oregon Press.

[B78] Kimmel CB, Ballard WW, Kimmel SR, Ullmann B, Schilling TF (1995). Stages of embryonic development of the zebrafish. Dev Dyn.

[B79] Lele Z, Engel S, Krone PH (1997). hsp47 and hsp70 gene expression is differentially regulated in a stress- and tissue-specific manner in zebrafish embryos. Dev Genet.

[B80] Lele Z, Folchert A, Concha M, Rauch GJ, Geisler R, Rosa F, Wilson SW, Hammerschmidt M, Bally-Cuif L (2002). parachute/n-cadherin is required for morphogenesis and maintained integrity of the zebrafish neural tube. Development.

